# Genomic Characterization of Circulating Dengue Virus, Ethiopia, 2022–2023

**DOI:** 10.3201/eid3103.240996

**Published:** 2025-03

**Authors:** Adugna Abera, Houriiyah Tegally, Geremew Tasew, Eduan Wilkinson, Abraham Ali, Feyisa Regasa, Molalegne Bitew, Mahlet Belachew, Lucious Chabuka, Gaspary Mwanyika, Derek Tshiabuila, Jennifer Giandhari, Sureshnee Pillay, Jenicca Poogavanan, Monika Moir, Moritz U.G. Kraemer, Kamran Khan, Carmen Huber, Getachew Tollera, Tobias F. Rinke de Wit, Cheryl Baxter, Richard Lessells, Dawit Wolday, Dereje Beyene, Tulio de Oliveira, CLIMADE Consortium

**Affiliations:** Ethiopian Public Health Institute, Addis Ababa, Ethiopia (A. Abera, G. Tasew, A. Ali, F. Regasa, M. Belachew, G. Tollera, D. Wolday); Addis Ababa University, Addis Ababa (A. Abera, D. Beyene); Centre for Epidemic Response and Innovation, School of Data Science and Computational Thinking, Stellenbosch University, Stellenbosch, South Africa (H. Tegally, E. Wilkinson, L. Chabuka, G. Mwanyika, D. Tshiabuila, J. Poogavanan, M. Moir, C. Baxter, T. de Oliveira); Bio and Emerging Technology Institute, Addis Ababa (M. Bitew); University of KwaZulu-Natal, Durban, South Africa (J. Giandhari, S. Pillay, R. Lessells, T. de Oliveira); University of Oxford, Oxford, UK (M.U.G. Kraemer); University of Toronto, Toronto, Ontario, Canada (K. Khan); BlueDot, Toronto (K. Khan, C. Huber); University of Amsterdam, Amsterdam, the Netherlands (T.F. Rinke de Wit); McMaster University, Hamilton, Ontario, Canada (D. Wolday)

**Keywords:** dengue, dengue virus, DENV, serotype, genotype, phylogenetic analysis, seasonality, human mobility, vector-borne infections, viruses, zoonoses, Ethiopia

## Abstract

In Ethiopia, dengue virus (DENV) infections have been reported in several regions; however, little is known about the genetic diversity of circulating viruses. We conducted clinical surveillance of DENV during the 2023 nationwide outbreak in Ethiopia. We enrolled patients at 3 sentinel hospital sites. Using reverse transcription PCR, we screened serum samples for 3 arboviruses and then serotyped and whole-genome sequenced DENV-positive samples. We detected DENV-1 and DENV-3 serotypes. Phylogenetic analysis identified 1 transmission cluster for DENV-1 (genotype III major lineage A) and 2 clusters for DENV-3 (genotype III major lineage B). The first DENV-3 cluster was closely related to an isolate from a 2023 dengue outbreak in Italy; the second cluster was related to isolates from India. Co-circulation of DENV-1 and DENV-3 in Ethiopia highlights the potential for severe dengue. Intensified surveillance and coordinated public health responses are needed to address the threat of severe dengue outbreaks.

Dengue virus (DENV) is primarily transmitted by *Aedes* spp. mosquitoes and causes considerable epidemics in tropical and subtropical regions. In 2023, the World Health Organization African Region reported 171,991 suspected cases, 70,223 of which were confirmed cases with 753 deaths (*1*,*2*). Outbreaks have been reported in 15 countries in Africa; Burkina Faso accounted for 85% of cases, followed by Ethiopia (8.2%), Mali (2.5%), and Côte d’Ivoire (2.2%) (*1*,*2*).

Ethiopia has had several outbreaks of dengue fever since 2013. The first recorded outbreak was reported in Dire Dawa and affected 9,441 persons (*3*). A second outbreak occurred in Godey (Somali Region) in 2014 and Afar Region in 2015 (*4*). Since 2015, an increase in the number of severe febrile illness cases has been observed in Godey, Dire Dawa, and Afar Region with no apparent cause. A dengue outbreak began in April 2023 in Afar Region, followed by Dire Dawa and the neighboring regions of Amhara and Oromia. A total of 27,577 cases and 21 deaths in 12 districts have been reported in the eastern part of Ethiopia (*1*,*5*).

DENV is a single-stranded, positive-sense RNA virus with 4 serotypes, DENV-1–4 (*6*,*7*). The risk of severe dengue increases after infections with different serotypes. Each serotype is further divided into several genotypes. However, different genotypes of the same serotype might vary in their ability to infect host cells and cause severe forms of disease. According to genetic characterization, DENV-1 has 5 defined genotypes, DENV-2 has 6 defined genotypes, DENV-3 has 5 defined genotypes, and DENV-4 has 4 defined genotypes (*8*–*11*). A nomenclature system has been recently proposed to further subdivide genotypes into major and minor lineages to aid global monitoring efforts (*12*).

During dengue outbreaks, the emergence of new virus serotypes or changes in circulating genotypes in a particular region can lead to more severe outbreaks (*13*,*14*). Because no approved medical countermeasures for treating severe dengue exist, little is known about the efficacy of dengue vaccines and potential antiviral drugs. Therefore, it is crucial to continuously monitor the genetic diversity of circulating DENVs in dengue-endemic areas. Surveillance will be instrumental in developing and evaluating vaccines and treatments (*15*) and responding effectively to dengue outbreaks.

Although multiple outbreaks and geographic expansion of DENV in Ethiopia have occurred, diversity of circulating DENVs in this country has not been characterized, and whole-genome sequences have not been publicly available. We addressed this information gap by identifying circulating DENV serotypes and genotypes among patients with febrile illness in 3 hospital-based sentinel sites in Ethiopia. Furthermore, we conducted phylogenetic analyses to determine spatial and temporal patterns of dengue transmission in Ethiopia. The study was approved by Addis Ababa University (approval no. CNCSDO/175/15/2023) and the Institutional Review Board of the Ethiopian Public Health Institute (EPHI; approval no. EPHI-IRB-536–2023).

## Methods

### Patient Characteristics and Study Settings

We conducted a cross-sectional hospital-based study at 3 major public hospitals in Dubti (Afar Region), Dire Dawa, and Gambela (Appendix Figure 1). Those hospitals serve as sentinel sites for acute febrile illnesses in Ethiopia and were selected because of their history monitoring arbovirus outbreaks and collecting serologic evidence for arboviruses (*5*,*16*). During December 2022–November 2023, we enrolled outpatients and inpatients who had fevers >37.5°C. We obtained informed consent from all participants >18 years of age. For persons 1–17 years of age, we obtained informed consent from a parent or guardian and assent from the child, when appropriate. 

### Eligibility/Exclusion Criteria

The inclusion criterion for the study was a fever of >37.5°C according to the 2009 World Health Organization dengue classification (*17*). We excluded patients with severe and established chronic clinical illnesses, such as persons living with HIV, malignancies, and known metabolic disorders.

### Sample Collection and Processing

At each study site, 5 mL of whole blood was collected from each participant into serum separator tubes. At the Afar site, blood samples were collected during April 25–May 15, 2023. Serum samples were isolated and collected in tubes containing 1 mL DNA/RNA Shield (Zymo Research, https://www.zymoresearch.com) and stored at −20°C during field collections. The field samples were transported in a cold chain to the EPHI laboratory, where we stored them at −20°C until PCR and sequencing analyses. The blood samples from Dire Dawa and Gambela sites were collected during June–October 2023; serum samples were isolated and stored at −20°C and were then shipped in a cold chain to the EPHI laboratory, where we stored them at −80°C until analysis.

### RNA Extraction and PCR Amplification

We performed RNA extraction at EPHI within 5 days of receiving the serum samples from each site by using a Bioer NPA-32P instrument (Bioer Technology, https://www.bioer.com.cn) according to the manufacturer’s instructions; final volume of RNA extract was 70 µL. We stored the remaining serum samples from Afar Region at −20°C for whole-genome sequencing and stored remaining samples from the Dire Dawa and Gambela sites at −80°C.

On the same day we extracted RNA, we used the US Centers for Disease Control and Prevention (CDC) Trioplex Real-Time RT-PCR Assay to screen for arboviral infections, including those caused by DENV, chikungunya virus (CHIKV), and Zika virus (ZIKV). This assay includes a set of published oligonucleotide primers and dual-labeled hydrolysis Taqman probes. In brief, we combined 10 µL of RNA sample with 12.5 µL of PCR master mix reaction buffer (Thermo Fisher Scientific, https://www.thermofisher.com), 1 µmol/L virus-specific primers, 0.3 µmol/L dengue-specific probe, 0.15 µmol/L CHIKV-specific probe, 0.15 µmol/L ZIKV-specific probe, and nuclease-free water in a 96-well optical PCR plate to make a final reaction volume of 25 µL. We captured the fluorescent signal intensity by using the QuantStudio 5 Real-Time PCR System (Thermo Fisher Scientific).

### DENV Serotyping

After PCR screening, we serotyped DENV-positive samples by using the same RNA extract and the CDC DENV-1-4 rRT-PCR Multiplex Assay on the QuantStudio 5 Real-Time PCR System. This assay uses specific primers and probes to detect DENV-1–4. To perform the assay, we mixed 5 µL of extracted RNA with 12.5 µL of master mix (Thermo Fisher Scientific), according to the manufacturer’s protocol, and DENV-1–4 primers and probes provided by CDC.

### DENV Sequencing

After serotyping, we selected specimens with PCR cycle thresholds (Cts) of <26 for sequencing. Technical support staff from KwaZulu-Natal Research Innovation and Sequencing Platform, University of KwaZulu-Natal, and Centre for Epidemic Response and Innovation, Stellenbosch University, performed sequencing. The primer scheme for DENV sequencing and other arboviruses is available on the CLIMADE GitHub (https://github.com/CERI-KRISP/CLIMADE/tree/master/Protocols/Arboviruses). We adapted the COVIDSeq protocol to perform the library preparation, followed by sequencing on the Illumina Miseq platform (Illumina, https://www.illumina.com). The sequencing protocol is also available on the CLIMADE GitHub (https://protocols.io/view/pathogen-whole-genome-sequencing-multiplexed-ampli-cgwbtxan).

We prepared libraries by using the Illumina COVIDSeq protocol. In brief, we reverse transcribed RNA to cDNA by using random hexamers and amplified the DENV genome by using 2 pools of primers specific for the DENV-1–4 serotypes. We used enrichment bead-linked transposomes (Illumina) to tagment PCR amplicons and further amplified adaptor-ligated amplicons by using the unique 10 bp Index 1 (i7) adapters and Index 2 (i5) adapters (Illumina-PCR Indexes Set 1; Illumina) for each sample. We quantified the pooled amplicon library by using a Qubit 2.0 fluorometer (Thermo Fisher Scientific) and diluted the library to 4 nmol/L. We then denatured the library and diluted it to a final loading concentration of 12 pmol/L. We performed dual indexed paired-end sequencing on an Illumina Miseq instrument with a v3 600-cycle flow cell.

### Bioinformatics Analysis

After base calling and demultiplexing of the sequence runs, we processed fastq files by using the Genome Detective version 2.13.3 (https://www.genomedetective.com) analysis pipeline. We retrieved consensus fasta and binary alignment map files for each sample from Genome Detective and performed sequence genotyping and lineage classification by using the Genome Detective Dengue Typing Tool according to a newly developed nomenclature for DENV classification (*12*). We submitted all genomic sequences to the GISAID Epi-Arbo database (https://www.gisaid.org; accession nos. EPI_ISL_19229161–193). 

### Sequence Alignment and Phylogenetic Analysis

We retrieved DENV reference sequence datasets from GenBank and the GISAID Epi-Arbo database and removed duplicate entries. We subjected the retrieved sequences to initial quality control by removing unverified sequences and incomplete records (i.e., geographic location and sampling dates). The final dataset used for phylogenetic analyses consisted of 33 sequences generated from Ethiopia as well as 2,348 publicly available sequences of DENV-3 genotype III major lineage B and 990 sequences of DENV-1 genotype III major lineage A, corresponding to transmission lineages detected in Ethiopia. We aligned the DENV-1 and DENV-3 datasets from this study with the appropriate DENV serotype reference genomes (DENV-1, GenBank accession no. NC_001477.1; DENV-3, accession no. NC_001475.2) by using the Nextalign version 1.3.0 alignment tool (*18*).

We generated maximum-likelihood trees for each of the 2 genotypes by using IQ-TREE version 2.2.2.2 and 1,000 bootstraps (*19*). Using ModelFinder in IQ-TREE, we selected the following nucleotide substitution models: transition 2 plus base frequencies plus proportion of invariable sites plus gamma distribution 4 model for DENV-1 and the general time reversible plus base frequencies plus proportion of invariable sites plus gamma distribution 4 model for DENV-3, according to Bayesian information criterion (*19*). We evaluated the molecular clock signal by using TempEst v1.5.3 (*20*) and removed potential outliers that violated the molecular clock assumption before inferring a time-scaled tree by using TreeTime version 0.10.0 (*21*). We used molecular clock rates of 5.015 × 10^−4^ (for DENV-1) and 1.225 × 10^−4^ (for DENV-3) nucleotide substitutions per site per year, determined by the clock function in TreeTime.

### Time-Calibrated Bayesian Phylogenies

We constructed time-calibrated Bayesian phylogenetic trees to estimate the time to the most common recent ancestor (tMRCA) (i.e., time of emergence) of the DENV lineages circulating during the 2023 outbreak and likely introduction routes. We used BEAST version 1.10.4 along with the BEAGLE library version 3.2.0 to improve computational speed (*22*,*23*). We applied a relaxed clock model for all analyses along with the Hasegawa-Kishino-Yano plus gamma distribution plus proportion of invariable sites nucleotide substitution model and used a constant population coalescent model assumption. Subsequently, we performed all analyses in 2 independent runs with 100 million iterations each. We checked convergence of Markov chain Monte Carlo chains by using Tracer version 1.7.1 (*24*) and discarded 10% of initial chains as burn-ins. We pooled postburn-in samples to summarize parameter estimates by using LogCombiner and TreeAnnotator tools in BEAST, including posterior probability for each parameter and maximum-clade credibility trees. We visualized phylogenetic trees by using Figtree version 1.4.4 (http://tree.bio.ed.ac.uk/software/figtree) and other figures by using R (The R Project for Statistical Computing, https://www.r-project.org) and ggplot (*25*).

### Air Travel Data

We analyzed travel data from the International Air Transport Association (https://www.iata.org) to determine the volume of passengers arriving from international airports. Those data accounted for ≈90% of passenger travel itineraries on commercial flights, excluding transportation via unscheduled charter flights. The remaining 10% of commercial flights is modeled by using market intelligence.

## Results

**Figure 1:**
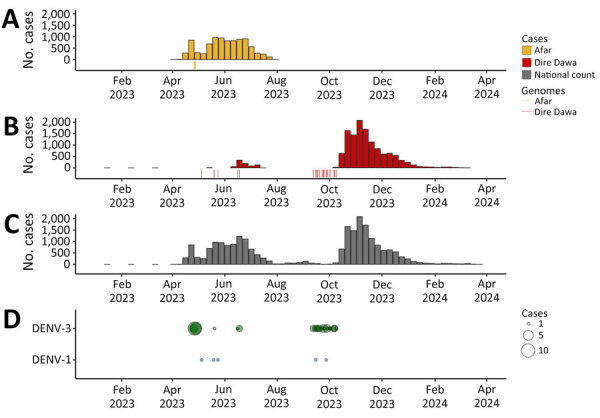
Spatiotemporal distribution of dengue cases in study of genomic characterization of circulating DENV, Ethiopia, 2022–2023. A–C) Number of dengue cases in Ethiopia in Afar Region during April 2023–August 2023 outbreak (A), Dire Dawa during June 2023–April 2024 outbreak (B), and national count during 2023–2024 (C). Each colored vertical line under bars indicates 1 sequenced genome. D) DENV serotype distribution of sampled cases. Size of circles indicates number of genotyped cases for each serotype. DENV, dengue virus.

**Figure 2:**
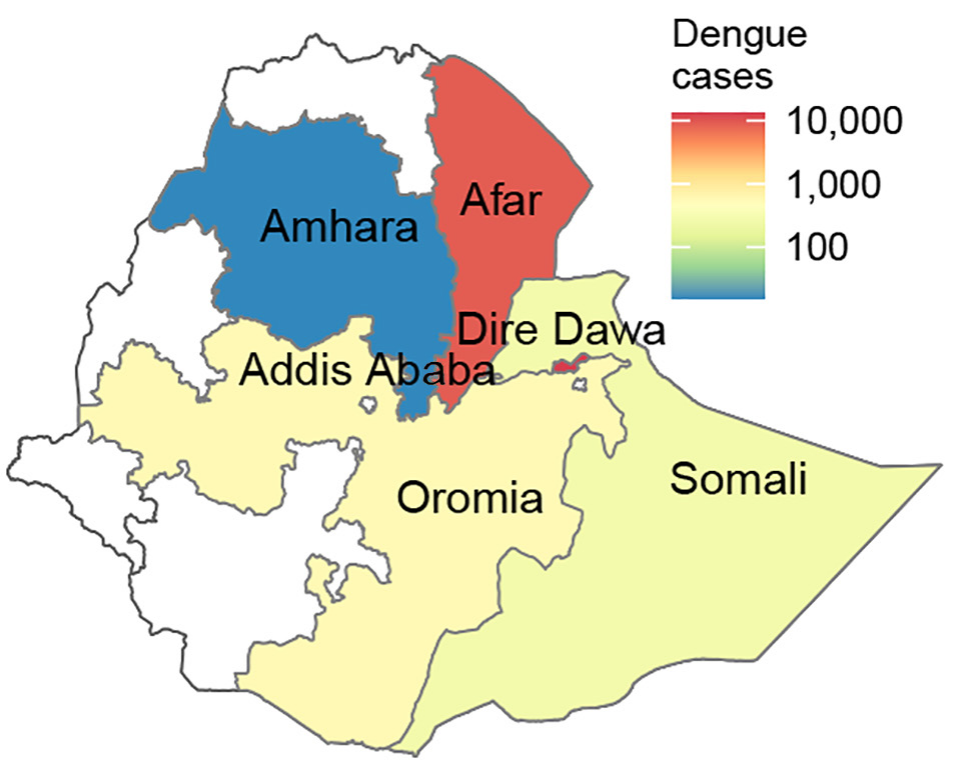
Number of dengue cases in different regions of Ethiopia during 2023–2024 in study of genomic characterization of circulating dengue virus. Only dengue virus (DENV) serotype 3 was found in Afar Region; both DENV-1 and DENV-3 were isolated in the city of Dire Dawa.

We recruited study participants during a recorded DENV infection outbreak in Ethiopia (Appendix Figure 1). Cases were recorded in multiple regions of the country by the Ministry of Health in Ethiopia starting in early 2023; Afar Region and Dire Dawa were the 2 most affected regions (Figures 1, 2). A first peak in cases occurred in Afar Region during April–August 2023; low level transmission occurred in Dire Dawa during that period. A second prominent peak was observed in Dire Dawa during October 2023–January 2024. Our clinical surveillance was able to detect acute dengue infections from both regions; detection in Dire Dawa intensified before the peak in recorded cases (Figure 1).

### Sample Collection and Epidemiology

**Figure 3:**
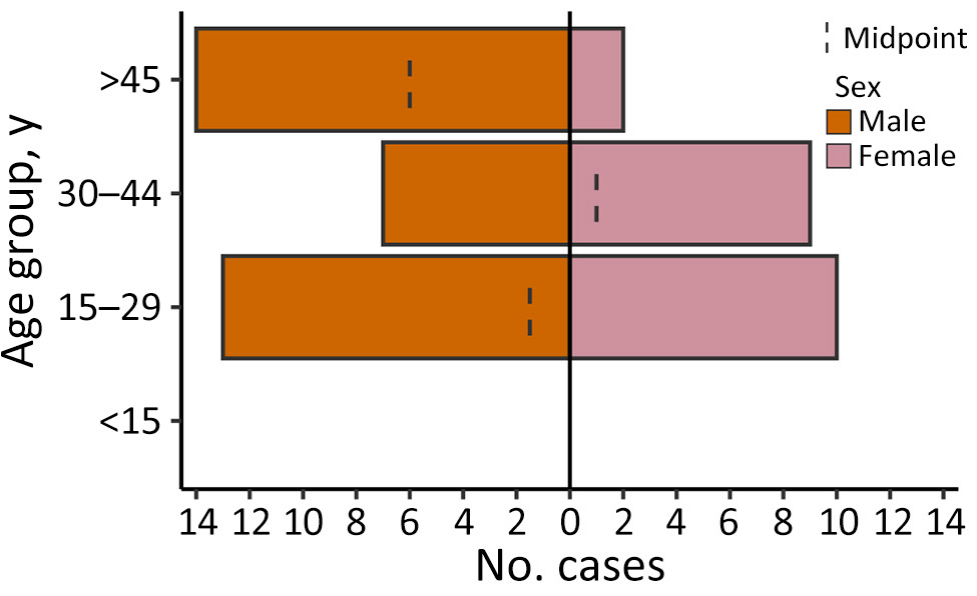
Demographic distribution of patients with dengue virus infections in study of genomic characterization of circulating dengue virus, Ethiopia, 2022–2023.

Of 891 febrile patients screened for DENV, CHIKV, and ZIKV infections, only DENV was detected. The percentage of patients with a positive PCR for DENV was 10.4% (93/891). The test positivity was slightly higher in men than women (Table; Figure 3). DENV was isolated from 2 of the 3 sites; the percentages of patients with a positive DENV PCR were 13.7% (41/300) in Afar Region and 17.9% (52/291) in Dire Dawa. None of the 300 samples from Gambela were positive for DENV. Virus genotyping showed evidence of co-circulation of DENV-1 and DENV-3 throughout the outbreak period (Figure 1, panel D). Among 93 PCR-positive isolates, we identified 88 (94.6%) as DENV-3 and 5 (5.4%) as DENV-1. At the Afar site, only DENV-3 was detected (n = 41), whereas in Dire Dawa, both DENV-1 (n = 5) and DENV-3 (n = 47) were detected (Table).

**Table:** Characteristics of patients with DENV infections in study of genomic characterization of circulating dengue virus, Ethiopia, 2022–2023*

Parameters	No. dengue tests		Total no. tests	% Positive tests	DENV serotypes, no. patients		
	Positive	Negative			DENV-1	DENV-3	Total
Sex							
M	49	400	449	10.91	2	47	49
F	44	398	442	9.95	3	41	44
Region							
Afar	41	259	300	13.67	0	41	41
Dire Dawa	52	239	291	17.87	5	47	52
Gambela	0	300	300	0	0	0	0
Age, y							
0–4	0	27	27	0	0	0	0
5–14	2	40	42	4.8	0	2	2
15–29	41	393	434	9.4	2	40	42
30–44	28	228	256	10.9	1	27	28
>45	21	111	132	15.9	2	19	21

*DENV, dengue virus.

### Virus Genome Sequencing

**Figure 4:**
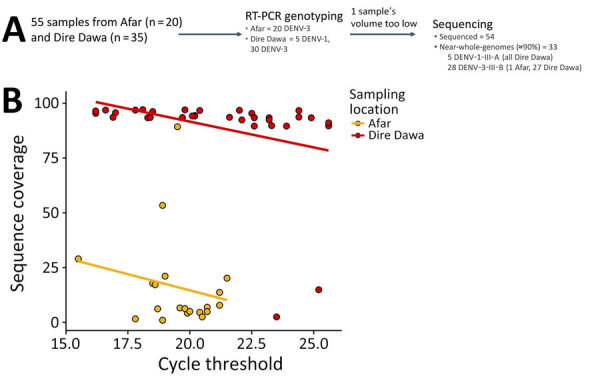
Sequencing process and coverage results in study of genomic characterization of circulating DENV, Ethiopia, 2022–2023. A) Patient sample selection, genotyping, and sequencing workflow. B) PCR cycle thresholds compared with sequence coverage for all sequenced specimens from Afar Regions and Dire Dawa. DENV, dengue virus; III-A, genotype III lineage A; III-B, genotype III lineage B; RT-PCR, reverse transcription PCR.

We selected 55 virus isolates for sequencing on the basis of their PCR Cts. Cts ranged from 15.5 to 25.6. We selected 20 DENV-3 isolates from Afar Region and 35 (5 DENV-1 and 30 DENV-3) from Dire Dawa for sequencing. Of those, 54 isolates were successfully sequenced; 1 sample failed to be sequenced because of low sample volume. Among the successfully sequenced isolates, 33 gave near whole-genome sequences (>90% genome coverage). Of those 33, we identified all 5 DENV-1 as DENV-1 genotype III lineage A and the remaining 28 as DENV-3 genotype III lineage B (1 isolate from Afar Region and 27 from Dire Dawa) (Figure 4).

Samples collected from Dire Dawa showed an expected association between a high virus load (lower Ct values) and high sequence coverage, whereas most samples from Afar Region produced low sequence coverage genomes, despite low Cts. PCR was not repeated after storing the serum samples; we only used Cts from the first screening PCR. Sample collection and storage freezers used at both sites were different. The samples from Afar Region were collected in DNA/RNA Shield during the outbreak in May 2023 and stored for a long period at −20°C, during which intermittent temperature fluctuations of the freezer were noted. In contrast, samples collected from Dire Dawa and Gambela were relatively recent and stored at −80°C until they were sequenced.

### Phylogenetic Analysis 

**Figure 5:**
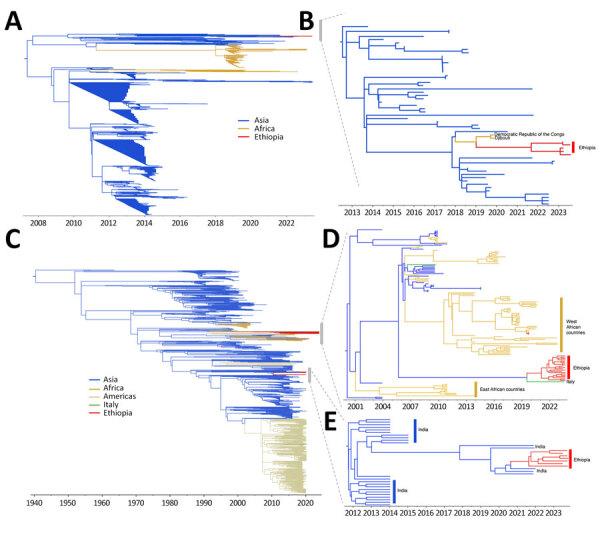
Time-scaled phylogenetic analysis of genomes from Ethiopia in study of genomic characterization of circulating dengue virus (DENV), 2022–2023. A) Time-scaled maximum-likelihood phylogeny of DENV-1 genotype III major lineage A clade sequences containing sequenced genomes from this study. B) Time-scaled phylogeny of subclade of tree in panel A, indicating close evolutionary relationships of DENV-1 sequences from Ethiopia. C) Time-scaled maximum-likelihood phylogeny of DENV-3 genotype III major lineage B clade sequences containing sequenced genomes from this study. D) Time-scaled maximum clade credibility tree of subclade from tree in panel C indicating phylogenetic relationships of cluster 1 of DENV-3 genomes from Ethiopia. E) Time-scaled maximum clade credibility tree of subclade from tree in panel C indicating phylogenetic relationships of cluster 2 of DENV-3 genomes from Ethiopia. Colors indicate country or continent origin of sequences used in trees.

We constructed a time-scaled phylogenetic tree by aligning 990 global DENV-1 genotype III lineage A sequences along with 5 sequences from Ethiopia obtained in this study (Figure 5, panel A). For this lineage, all sequences from Ethiopia belonged to a single transmission cluster, which clustered monophyletically with other sequences from Africa, suggesting cryptic transmission in the region since 2019 (Figure 5, panel B). tMRCA indicated that emergence of the clade from Ethiopia occurred during mid-2021 (Figure 5, panel B). Sequences originating mostly from Asia are basal to the clade from Africa, suggesting an introduction into Africa from Asia in 2018 (Figure 5, panel B). For DENV-3 genotype III lineage B, we constructed a time-scaled phylogenetic tree by using 2,348 global sequences along with sequences from Ethiopia (Figure 5, panel C). Those sequences from Ethiopia belonged to 2 distinct clades (Figure 5, panels D, E). Whereas the first clade consisted of sequences generally from Asia and Africa, sequences from this study had tMRCA in early 2021 (95% highest posterior density [HPD] 2020–2022) and clustered monophyletically with a sequence from Italy’s 2023 dengue outbreak (*26*). We infer that the common ancestor for the isolates from Ethiopia and Italy existed around mid-2019 (95% HPD mid-2017–2021) with long branches separating the 2 locations. The Ethiopia/Italy clade was supported with >70% posterior support at the relevant internal nodes on the maximum-clade credibility tree, suggesting some level of cryptic transmission in unsampled areas where both Ethiopia and Italy could have received virus introductions. However, given historical ties between the 2 countries and continued high connectivity, an introduction into Ethiopia from Italy is plausible; Italy has the second highest number of air travel passengers into Ethiopia of all countries within Europe (Appendix Figure 2). The second relevant clade of this lineage indicates a clear introduction from Asia into Ethiopia in late 2021 (95% HPD 2021–2023) and a potentially persistent transmission since then (Figure 3, panel E). Although the closest relatives to this second clade from Ethiopia are sequences from India, it remains possible that intermediate unsampled regions are involved because of the long branch lengths on the tree, including Saudi Arabia (*27*), which has the second largest air travel passenger volume into Ethiopia overall (Appendix Figure 2).

## Discussion

We investigated the genomic epidemiology of DENV in 3 areas of Ethiopia and determined the distribution of serotypes, genotypes, and lineages circulating in Ethiopia, identified by whole-genome sequencing. Our findings showed that 2 serotypes, DENV-1 and DENV-3, were isolated in Dire Dawa, whereas only DENV-3 was isolated in Afar Region. Those data suggest a need for more comprehensive epidemiologic or genomic surveillance and strengthened systems for severe dengue surveillance because of the potential health risks from multiple serotypes or genotypes. In Africa, all 4 DENV serotypes have been detected in both humans and *Aedes* spp. mosquitoes (*28*). DENV-2 is the most prevalent serotype in East Africa. The virus caused outbreaks in Ethiopia in 2013, Kenya in 2013, Tanzania in 2014, and Mozambique in 2014–2015 and has remained prevalent in those areas (*29*–*31*). DENV-1 infection outbreaks have been detected at different times in Angola, Kenya, Senegal, and Somalia (*32*). The current DENV-1 serotype might have been historically co-circulating with DENV-2 or might have been a recent introduction into Dire Dawa. The current dengue fever outbreak in Ethiopia first began in the Mile district of Afar Region in April 2023; the outbreak was caused by DENV-3 and has since spread to >5 other regions in the eastern part of the country (*33*).

Phylogenetic analysis revealed that DENV-1 isolates from this study belong to genotype III major lineage A; circulation of this lineage during the 2023 outbreak corresponds to a single transmission cluster. It seems that this cluster was introduced into Ethiopia from Asia via other countries in Africa. DENVs belonging to the same genotype were sequenced during the 2023 dengue outbreak in Chad (*2*). However, the genomes generated in this study are not directly linked to the Chad outbreak, which originated from a large outbreak in Tanzania in 2019 via Nigeria (*34*). The phylogenetic reconstruction from available DENV-1 genomic data in Africa, which we have supplemented with our sequences, indicate several distinct lineages are currently circulating on the continent. All known DENV-1 strains from Central Africa belong to genotype V African lineage; the oldest strains of this lineage were isolated in Nigeria in 1968 (*35*). However, the lack of publicly available data limits our understanding of the dynamics of DENV-1 within Africa. 

DENV-3 viruses sequenced during Ethiopia’s 2023 dengue outbreak belonged to genotype III major lineage B, and phylogenetic reconstructions revealed 2 transmission clusters circulating in Ethiopia. Cluster 1 is closely related to a DENV-3 from Italy sequenced in 2023 (Figure 5, panel D) (*28*), highlighting the possibility that the virus might have been introduced into Ethiopia from Italy. Because we have no previous sequence information, we cannot exclude the possibility that transmission from Ethiopia to Italy might also have occurred. DENV-3 genotype III exists in neighboring countries, including Sudan, Kenya, Djibouti, and Somalia (*36*). However, the current DENV-3 genotype III in Ethiopia is not directly related to the other isolates from Africa. Cluster 2 appears to have been introduced from India perhaps via secondary locations (Figure 5, panel E). Phylogenetic analysis of DENV-3 in India has shown that outbreaks during 2017–2018 were caused by genotype III (*37*).

By whole-genome sequencing, only 33 samples from this study resulted in >90% genome coverage. Thirty-two (5 DENV-1 and 27 DENV-3) of those samples were from Dire Dawa, but only 1 sample gave ≈90% genome coverage from Afar Region. Serum samples from Afar Region were collected during the outbreak and sent to the EPHI laboratory for DENV detection and serotyping. The remaining samples were then stored at −20°C for ≈11 months before sequencing. The limited genome coverage in some samples could be attributed to extended storage under unsuitable conditions. In contrast, serum samples obtained from Dire Dawa were stored at −80°C for ≈4–8 months, which likely resulted in greater sequence coverage.

The first limitation of our study is that poor sequencing outcomes were encountered, possibly because of inadequate sample storage in Afar Region. Improved sample storage conditions could have led to better whole-genome sequencing results. Second, the limited availability of DENV sequences continentally and globally restricted our ability to infer global transmission dynamics and direct introduction routes. However, we were able to identify circulating genotypes and lineages and infer possible introduction routes and timing. 

In conclusion, we characterized DENV genomic epidemiology during the 2023 dengue outbreak in Ethiopia. Our findings highlight the utility of comprehensive disease surveillance, including pathogen genome sequencing, to elucidate DENV transmission dynamics in Ethiopia and elsewhere during emerging outbreaks. Regular active surveillance of dengue through highly connected sentinel sites as well as at ports of entry can improve response time and reduce the likelihood of dengue outbreaks.

Appendix Additional information for genomic characterization of circulating dengue virus, Ethiopia, 2022–2023.
