# Supplementary material for: Genomic Characterization of Circulating Dengue Virus, Ethiopia, 2022–2023
Source: Emerg Infect Dis. 2025 Mar;31(3):516–25. doi: 10.3201/eid3103.240996 (PMC11878302; doi:10.3201/eid3103.240996)
Supplement: Appendix — Additional information for genomic characterization of circulating dengue virus, Ethiopia, 2022–2023. [file 24-0996-Techapp-s1.pdf]

Article DOI: https://doi.org/10.3201/eid3103.240996
EID cannot ensure accessibility for supplementary materials supplied by authors. Readers who have difficulty accessing supplementary content should contact the authors for assistance.

Genomic Characterization of Circulating Dengue Virus, Ethiopia, 2022–2023

Appendix

CLIMADE Consortium contributing authors:

Luiz C J Alcantara[5,6], Marta Giovanetti[5,6], Edward C Holmes[6], Vagner Fonseca[7], Tanya Golubchi[6], Samuel Oyola[8],, Jenicca Poongavanan[1], Graeme Dor[1], Gaspary Mwanyika[1], José Lourenco[10], Frank Tanser[1], Richard Lessells[2], Abdou Padane[11,] Ambroise Ahouidi[11], Abdualmoniem O A Musa[12], Adugna Abera[13], Allan Campbell[14], Aloysious S Semaganda[15], Argentina F Muianga[16], Bernard Onoja[17], Birhanu D Alemu[18], Darren Martin[19], Mohamed Z Alimohamed[20], Fredy B N Simo[21], Girma Godebo[22], James Ayei Maror[23,] John Oludele[24], Joseph Fokam[25], Kenneth K Maeka[26], Lavanya Singh[2], Martin Faye[27], Michael Owusu[28], Michel N Dikongo[29], Molalegne Bitew [30], Nkuurunziza Jerome[31], Nokuzola Mbhele[19], Oyewale Tomori[32], Ramuth Magalutcheemee[33], Sara A Abuelmaali[34], Wolfgang Preiser[35]

Author affiliations

[1]Centre for Epidemic Response Innovation (CERI), School for Data Science and Computational Thinking, Stellenbosch University, South Africa

[2]KwaZulu-Natal Research Innovation and Sequencing Platform (KRISP), University of KwaZulu-Natal, South Africa

[3]Duke Human Vaccine Institute, Duke University, Durham, NC 27710, USA

[4]Institute of Social and Preventive Medicine (ISPM), University in Bern, Switzerland

[5]Laboratório de Flavivírus, Instituto Oswaldo Cruz, Fundação Oswaldo Cruz, Rio de Janeiro, Brazil

[6]Instituto Rene Rachou, Fundação Oswaldo Cruz, Belo Horizonte, Minas Gerais, Brazil

[7]Marie Bashir Institute for Infectious Diseases and Biosecurity, School of Life and Environmental Sciences and School of Medical Sciences, University of Sydney, Sydney, NSW, Australia

[8]Organização Pan-Americana da Saúde/Organização Mundial da Saúde, Brasília, Distrito Federal, Brazil.8. Organização Pan-Americana da Saúde/Organização Mundial da Saúde, Brasília, Distrito Federal, Brazil

[9]International Livestock Research Institute (ILRI), Kenya

[10]CBR (Biomedical Research Centre), Universidade Católica Portuguesa, Oeiras, Portugal

[11]Institute de Recherche en Santé, de Surveillance Épidémiologique et de Formations (IRESSEF), Senegal

[12]General Administration of Laboratories and Blood Banks, Ministry of Health, Kassala state, Sudan

[13]Ethiopian Public Health Institute, Ethiopia

[14]Central Public Health Reference Laboratory, Sierra Leone

[15]National Health Laboratories and Diagnostic Services - Central Public Health Laboratories, Uganda

[16]Instituto Nacional de Saude, Mozambique), Aziza John Samson, Tanzania

[17]University of Ibadan, Nigeria

[18]PATH, Ethiopia

[19]University of Cape Town, South Africa

[20]Muhimbili University of Health and Allied Sciences, Tanzania

[21]Centre for Research in Infectious Disease, Cameroon

[22]Wachemo University, Ethiopia

[23]National Public Health Laboratory, South Sudan

[24]Instituto Nacional de Saude, Mozambique

[25]Chantal BIYA International Reference Centre (CIRCB), Cameroon

[26]National Microbiology Reference Laboratory, Ministry of Health, Zimbabwe

[27]Institute Pasteur de Dakar, Senegal

[28]Kwame Nkrumah University of Science and Technology, Ghana

[29]Biologiste, Gabon

[30]Bio and Emerging Technology Institute, Ethiopia

[31]Hope Africa University, National Institute of Public Health Reference Laboratory, Burundi

[32]African Centre of Excellence for Genomics of Infectious (ACEGID) Redeemer's University, Nigeria

[33]Ministry of Health, Mauritius

[34]National Public Health Laboratory, Sudan

[35]University of Stellenbosch/National Health Laboratory Service, Tygerberg, South Africa

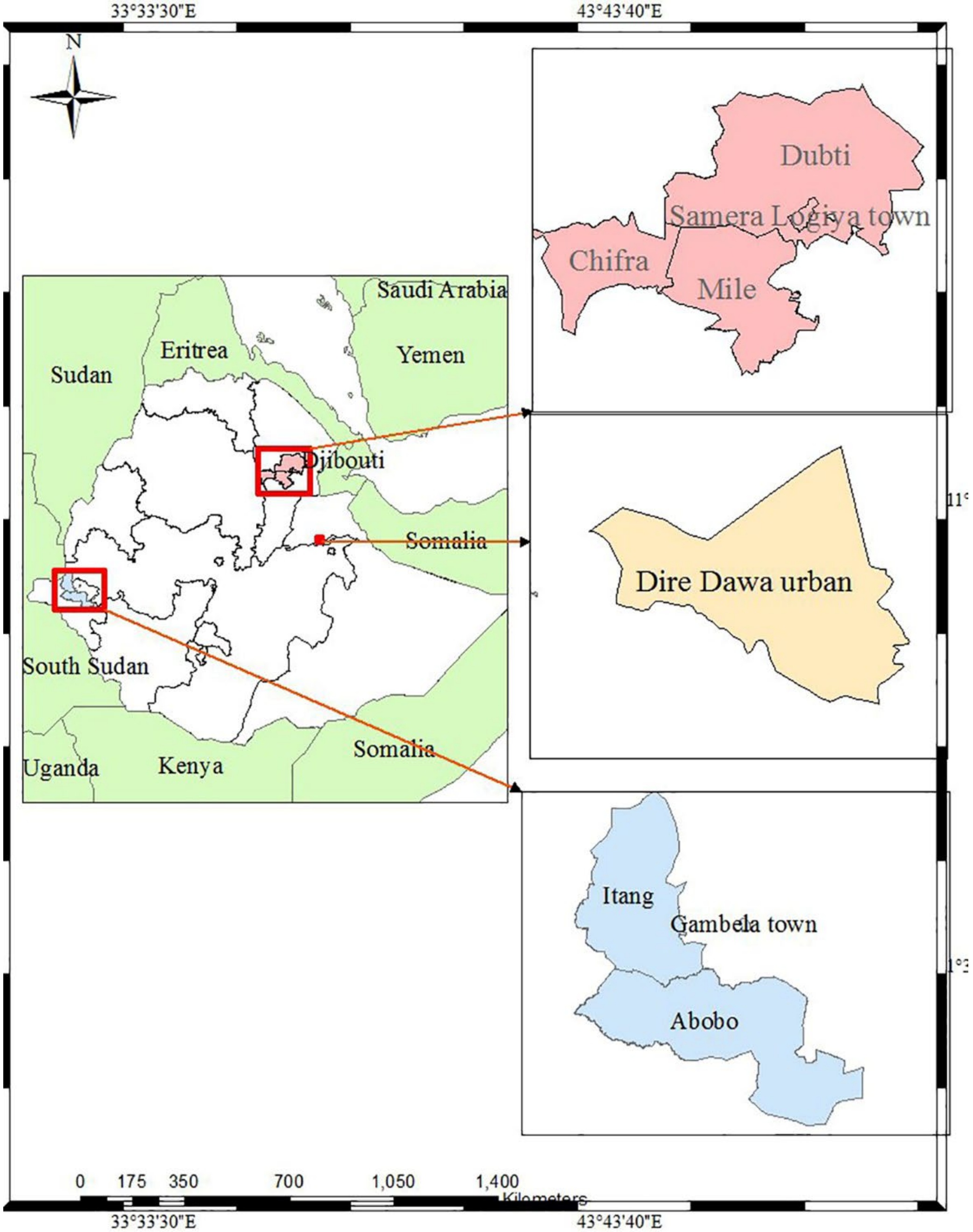

Appendix Figure 1. Map indicating the location of hospital-based sentinel sites in Ethiopia in study of genomic characterization of circulating dengue virus, 2022–2023. Map indicates 3 sentinel hospital sites where samples were collected from patients with febrile illness. Afar Region (in red) and Dire Dawa city administration are located in eastern Ethiopia, whereas the Gambela region is situated in the western Ethiopia.

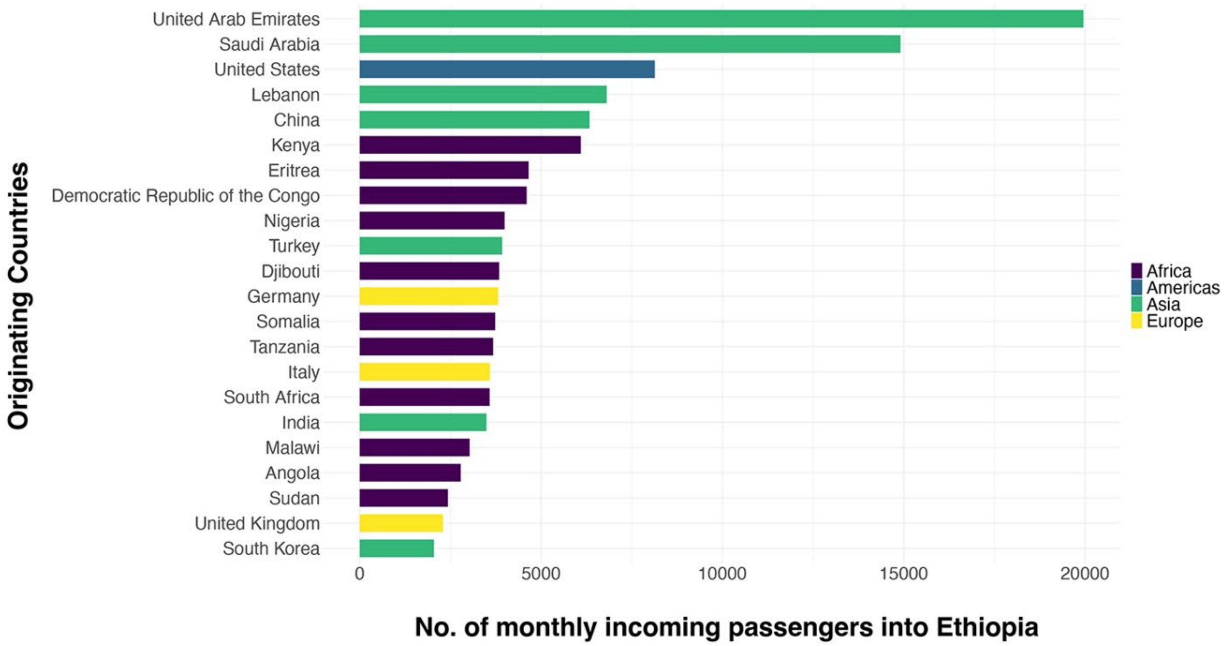

Appendix Figure 2. Example of monthly incoming air travel into Ethiopia in study of genomic characterization of circulating dengue virus, Ethiopia, 2022–2023. Countries with >2,000 incoming passengers are shown. Data are from the International Air Transport Association (https://www.iata.org).
